# Oxytocin Differentially Affects Sucrose Taking and Seeking in Male and Female Rats

**DOI:** 10.1016/j.bbr.2015.01.050

**Published:** 2015-01-31

**Authors:** Luyi Zhou, Shannon M. Ghee, Ronald E. See, Carmela M. Reichel

**Affiliations:** Department of Neurosciences, Medical University of South Carolina, Charleston, SC, 29425, USA

**Keywords:** female, oxytocin, reinstatement, sex differences, sucrose

## Abstract

Oxytocin has a modulatory role in natural and drug reward processes. While the role of oxytocin in pair bonding and reproduction has been extensively studied, sex differences in conditioned and unconditioned behavioral responses to oxytocin treatment have not been fully characterized. Here, we determined whether male and female rats would show similar dose response curves in response to acute oxytocin on measures of locomotor activity, sucrose seeking, and sucrose intake. Male and freely cycling female rats received vehicle or oxytocin (0.1, 0.3, 1, 3 mg/kg, IP) injections before behavioral tests designed to assess general motor activity, as well as sucrose self-administration and seeking. Lower doses of oxytocin decreased motor activity in a novel environment in females relative to males. Likewise, lower doses of oxytocin in females decreased responding for sucrose during maintenance of sucrose self-administration and reinstatement to sucrose-conditioned cues. However, sucrose seeking in response to a sucrose prime was only decreased by the highest oxytocin dose in both sexes. In general, oxytocin had similar effects in both sexes. However, females were more sensitive to lower doses of oxytocin than males. These findings are consistent with the notion that oxytocin regulates many of the same behaviors in males and females, but that the effects are typically more profound in females. Therapeutic use of oxytocin should include sex as a factor in determining dose regimens.

## 1. Introduction

Oxytocin is a classical and well-characterized neuroendocrine hormone that is a potent modulator of a variety of brain functions, including emotions, social interactions, and sexual behavior (1). In females, oxytocin has a critical role in reproduction as it induces uterine contractions during childbirth and facilitates milk ejection during lactation (2). Oxytocin is primarily synthesized in magnocellular neurons of the supraoptic and paraventricular nuclei of the hypothalamus and is secreted by axon terminals in the posterior pituitary into systemic blood circulation (3). In addition, oxytocin is produced in parvocellular and magnocellular neurons in the paraventricular nucleus that project to various brain regions and release oxytocin (4). Centrally acting oxytocin has a number of behavioral and physiological effects, including suppression of food intake (5). Specifically, peripheral injection of oxytocin in rats inhibits sucrose intake, whereas oxytocin receptor antagonists increase consumption (6, 7).

Although oxytocin's mediating role in food consumption has been previously studied, an area that has received little attention is in the regulation of appetitive-related behaviors. It is not surprising that oxytocin should have some impact on natural rewards like sucrose consumption, given its innervation of central appetitive pathways and modulation of drug reward (8, 9). Indeed, previous findings have shown that oxytocin knock-out mice displayed enhanced intake of sucrose solution (10) and oxytocin infusion into the ventral tegmental area suppressed sucrose intake (11). In an earlier study from our laboratory, systemic oxytocin (1 mg/kg) pretreatment attenuated sucrose seeking in a sucrose prime reinstatement test in both male and female rats; however, it did not impact motivation to lever press for a sucrose pellet at this dose (12).

In our previous study, males and females both exhibited oxytocin induced reductions in sucrose seeking. However, females show higher intake/preference of highly concentrated sweetened solutions than males (13). Additionally, when sweetened concentrations are reduced, ovariectomized females exhibited lower intake than males (14). Importantly, sex differences have been reported in the oxytocin system. For example, regardless of estrous cycle, females have significantly lower oxytocin receptor binding densities than males in the majority of forebrain regions involved in reward process (15).

Oxytocin also has anxiolytic effects in rats. In an open field test, a relatively low dose of oxytocin increased motor activity, whereas higher doses decreased activity in male rats (16). To this end, we first evaluated the dose response effects of oxytocin on novelty induced locomotor activity in male and female rats. Second, we studied the impact of oxytocin on operant sucrose self-administration in both sexes. Finally, we determined whether oxytocin would decrease reinstatement of sucrose seeking induced by either sucrose prime or conditioned cues.

## 2. Methods and Procedures

### 2.1. Subjects

A total of 91 male and female Sprague-Dawley rats (Charles River) were housed on a reversed 12:12 light-dark cycle in a temperature- and humidity-controlled vivarium (lights off at 06:00). Adult male rats weighed 275-300 g and adult females were 205-225 g at the time of delivery. Rats were individually housed and received *ad libitum* water and standard rat chow (Harlan, Indianapolis, IN, USA) until the locomotor test. After this test, subjects were food-restricted (≈20 g/day) to maintain 85% of *ad libitum* rats' body weight throughout the study. Procedures were conducted in accordance with the “Guide for the Care and Use of Laboratory Rats” (Institute of Laboratory Animal Resources on Life Sciences, National Research Council, 2011) and approved by the IACUC of the Medical University of South Carolina.

### 2.2. Locomotor Activity

To explore the effect of oxytocin on unconditioned locomotor activity in both males and females, rats underwent a single locomotor test. Locomotor activity was assessed in clear acrylic chambers (approximately 40×40×30 cm) equipped with Digiscan monitors (AccuScan Instruments Inc., Columbus, OH, USA). Each chamber contained a 16×16 photobeam array for the x and y axes and 16 photobeams for the z axis. Photobeam breaks were detected by a Digiscan analyser and recorded by DigiPro software (Version 1.4).

### 2.3. Sucrose taking, extinction, and reinstatement

Responding for sucrose was conducted in standard operant chambers (30×20×20 cm, Med Associates) housed inside sound-attenuating cubicles fitted with a fan for airflow and masking noise. Each chamber also contained two retractable levers, two stimulus lights, a speaker, and a house light. Rats were given daily 2 hr sessions to lever press for sucrose on a fixed ratio (FR) 1 schedule of reinforcement. During the sessions, a response on the active lever resulted in delivery of a sucrose pellet (45 mg, BioServe) combined with a 5 sec presentation of a light + tone stimulus complex, followed by an un-signaled 15 sec time out. Responses occurring during the time out and on the inactive lever were recorded without scheduled consequences.

Following sucrose self-administration, lever responding was extinguished in daily sessions, whereby operant responding no longer resulted in delivery of the sucrose reinforcement or cues. Extinction consisted of daily 2-hr sessions for at least 7 days and responding on either lever had no scheduled consequences. Extinction criterion was ≤ 20 presses for two consecutive days. When extinction criterion was met, behavior was reinstated by presentation of conditioned reinforcers (cued reinstatement) or by non-contingent sucrose delivery (primed reinstatement). During the cued reinstatement tests, active lever presses resulted in presentation of the light + tone stimulus in the same manner as during sucrose taking. During the sucrose prime tests, rats received one non-contingent pellet every 2 min for the first 10 min of the session and one pellet every 30 min thereafter, but responding on either lever had no scheduled consequences (12). Daily extinction sessions occurred for at least two days between reinstatement tests.

### 2.4. Estrous cycle monitoring

Females underwent daily post-session vaginal cytology procedures starting at least two days before the first test until the end of experiment for habituation purpose. Samples were collected with a sterile saline-dipped pipette tip and smeared onto glass slides, stained with Quik-Dip Hematology Stain (Mercedes Medical, FL), examined using a light microscope set at 10× magnification, and classified according to previously published criteria (17).

#### 2.4.1 The effect of oxytocin on locomotor activity

Males and females (n=8-11 per group) were injected IP with vehicle or one dose of oxytocin (0.1, 0.3, 1, or 3 mg/kg) at the volume of 1 ml/kg. Oxytocin was purchased from Cell Sciences (Canton, MA) and dissolved in ddH20. Following injection, rats were placed into their home cage for 30 min, and then placed in the locomotor chamber for an additional 90 min.

#### 2.4.2 The effect of oxytocin on sucrose intake

In this experiment, we tested the effects of oxytocin administration on established sucrose maintained responding using an FR1 schedule of reinforcement. Males and females rats (n=9-10) first learned to lever press for sucrose for at least 7 days (with ≥10 pellets/session). Once sucrose intake stabilized (within 20% difference in pellets received between the last two days), each rat was tested with a unique order of vehicle, 0.1, 0.3, 1 and 3 mg/kg oxytocin (IP). Each solution was administered 30 min before daily sucrose sessions. To reach criteria between tests, rats were required to have two consecutive days in which the numbers of pellets earned were within 20% of each other.

#### 2.4.3 The effect of oxytocin on sucrose conditioned cue and primed reinstatement

Males (n=21) and females (n=18) were first trained to lever press for sucrose on an FR1 schedule of reinforcement for 10 days, followed by at least 7 sessions of extinction. When extinction criterion was met, all rats were divided into two groups and underwent either cue-induced or sucrose-primed reinstatement. Before each discrete reinstatement trial, with a minimum of 2 extinction sessions between reinstatement tests, rats received an injection of oxytocin (0.1, 0.3, or 1 mg/kg) or vehicle in a counterbalanced order for a total of four reinstatement tests. Our laboratory has consistently shown that responding maintains stability over multiple reinstatement tests (18, 19) (20).

### 2.5 Data analysis

The primary dependent measures were distance traveled (Experiment 1), pellets delivered, and lever responses (Experiments 2 and 3). Males and females were directly compared with between or mixed 2-way analysis of variance (21), with sex as the between subjects factor and oxytocin dose as the within subjects factor. In Experiment 1, oxytocin dose was also a between subjects variable. Planned analyses of simple effects were conducted with Dunnett's multiple comparisons separately for males and females comparing each dose of oxytocin to vehicle. Significance was set at p<0.05 for all tests and all data are represented as the mean ± standard error of the mean (SEM). Females were freely cycling and tests were conducted based on a response criterion, rather than specific estrous cycle phase, resulting in an unequal distribution of females in each cycle phase. As such, including cycle in the analysis was not possible.

## 3. Results

### 3.1 The effect of oxytocin on locomotor activity

**Figure 1:**
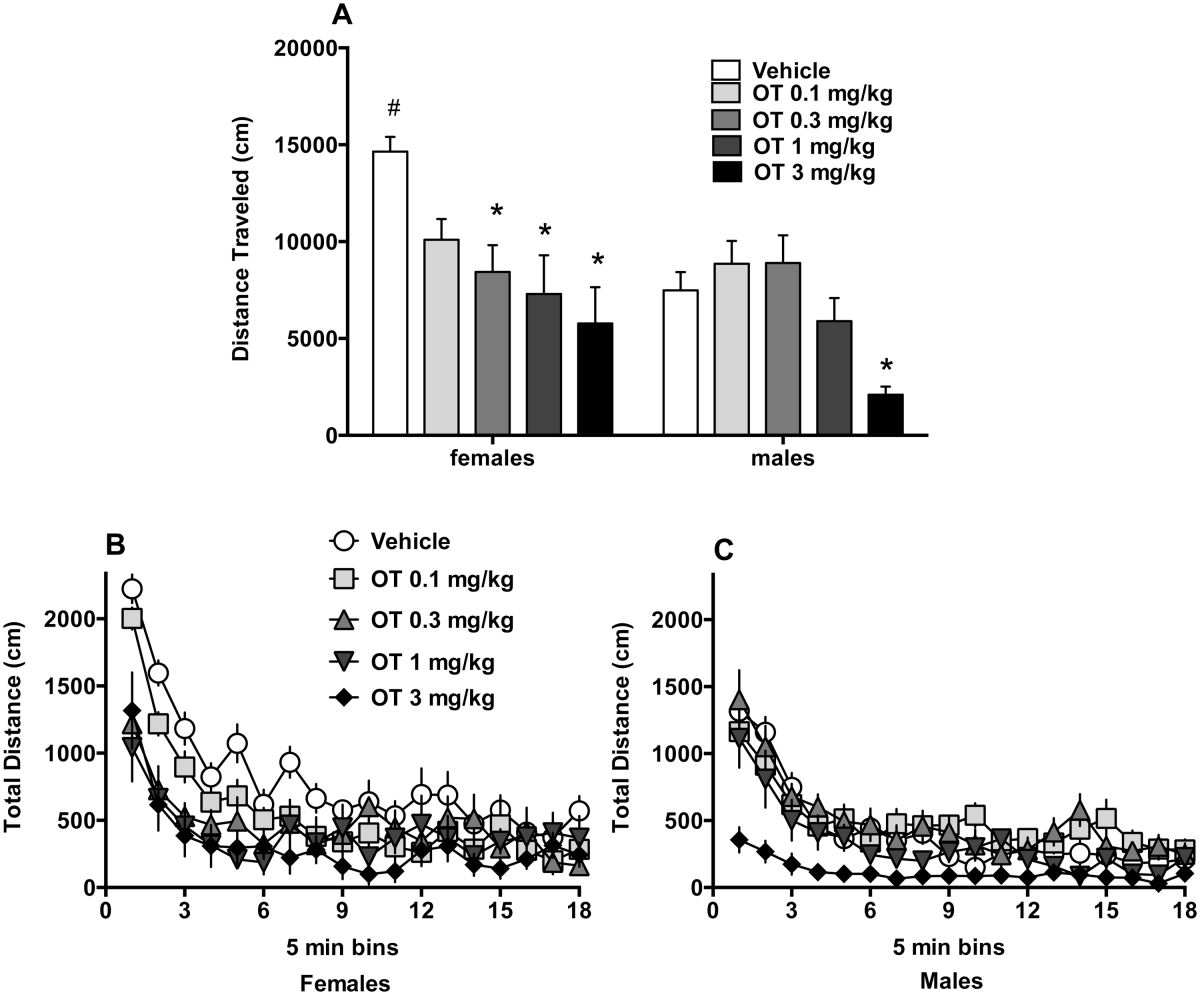
Novelty induced locomotor activity in male and female rats following oxytocin pretreatment (30 min injection to placement). A) Total distance traveled over 90 min session. Females had higher distance traveled scores that were reduced following oxytocin. B) Female and C) male distance traveled scores represented in 5 min time bins throughout the 90 min session. #Females significantly higher than male vehicle (p<0.05), *Significantly lower than same sex vehicle (p<0.05)

Figure 1A depicts sex differences in oxytocin's ability to decrease locomotor activity (i.e., distance traveled) over a 90 min period. Specifically, there was a sex × oxytocin dose interaction [F(4,81)=2.64, p<0.05], indicating that females were more prone to reduced locomotor activity than males. In females, three of the tested oxytocin (0.3, 1, and 3 mg/kg) doses decreased distanced traveled relative to vehicle (Dunnett's multiple comparisons, p<0.05). In contrast, only the highest oxytocin dose (3 mg/kg) decreased responding in males (p<0.05). Consistent with previous reports (22-24), vehicle-treated females had greater distance traveled scores than males in the novel environment (p<0.05). Not surprisingly, the same pattern occurred when evaluating distance traveled in 5 min time bins. For females (Figure 1B), there was a significant time × oxytocin dose interaction [F(68,595)=2.5, p<0.0001], with 0.3, 1, and 3 mg/kg oxytocin decreasing distance traveled across the session. For males (Figure 1C), there was also a significant time × oxytocin dose interaction [F(68,782)=2.3, p<0.0001], but only the high dose of 3 mg/kg decreased activity across the session.

### 3.2 The effect of oxytocin on sucrose intake

**Figure 2:**
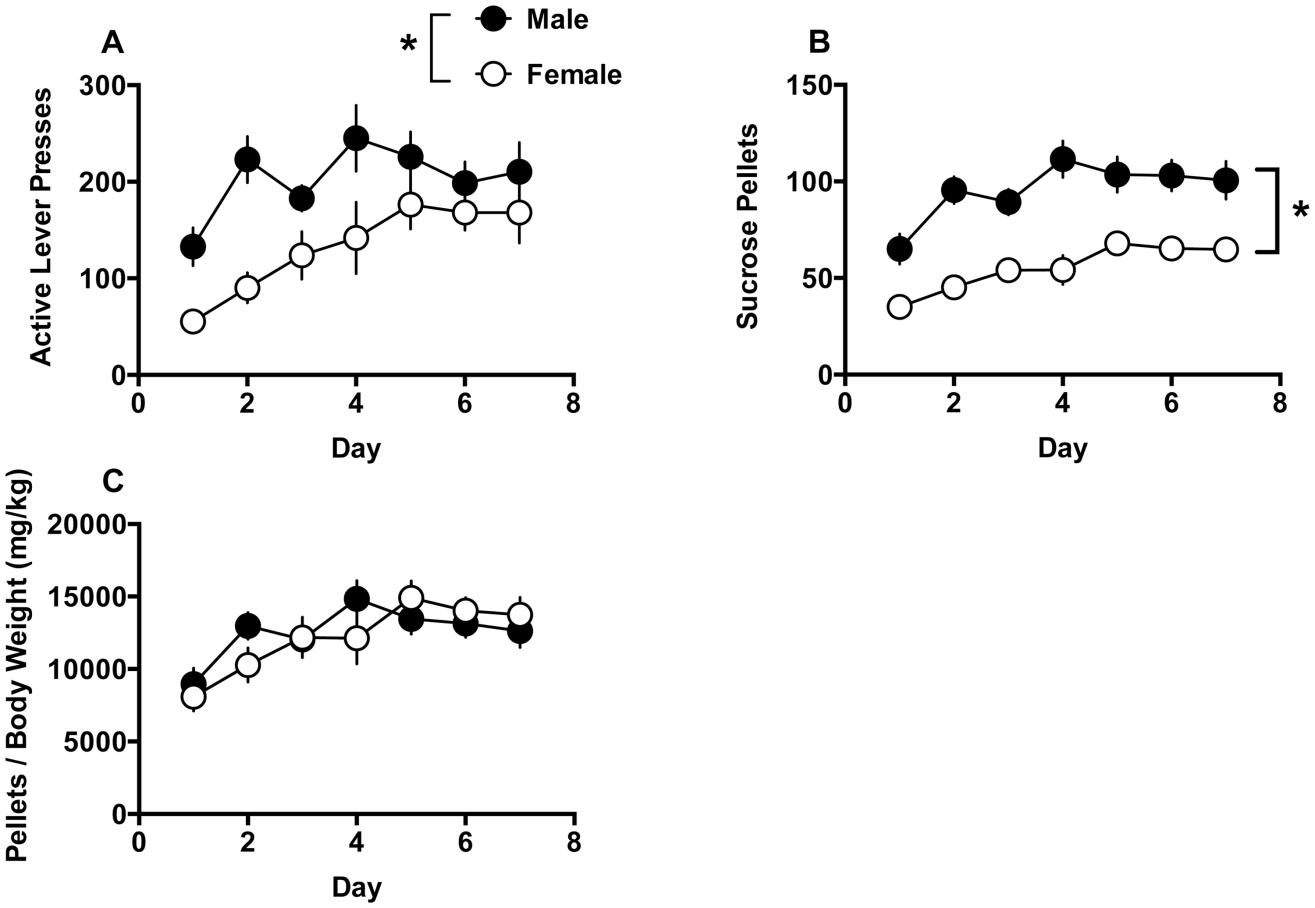
Lever presses and sucrose pellets earned during acquisition of sucrose responding. A) Lever presses during 7 days of sucrose availability. Males pressed the active lever more than females during this period. B) Sucrose pellets earned. Males earned more pellets than females. C) Adjusted pellets earned per body weight. Males and females did not differ during this time point on the pellets earned per body weight. *Significant difference from males (p<0.05)

In this experiment, sucrose self-administration was conducted as described above. During the 7 days of acquisition (Figure 2A), males made more responses on the active lever relative to females (sex main effect, F(1,17)=6.5, p<0.05), but responding was the same on the inactive lever (not shown). Males also received more pellets than females over the acquisition period (Figure 2B, sex main effect, F(1,17)=26.3, p<0.05); however, this difference was eliminated when body size was taken into account. Females are substantially smaller than males, so to better assess the sex differences in pellets earned, we calculated the amount of sucrose delivered to each rat with the following formula: mg sucrose/kg body weight. There were no differences between males and females in mg sucrose/kg body weight of sucrose that was delivered (Figure 2C).

**Figure 3:**
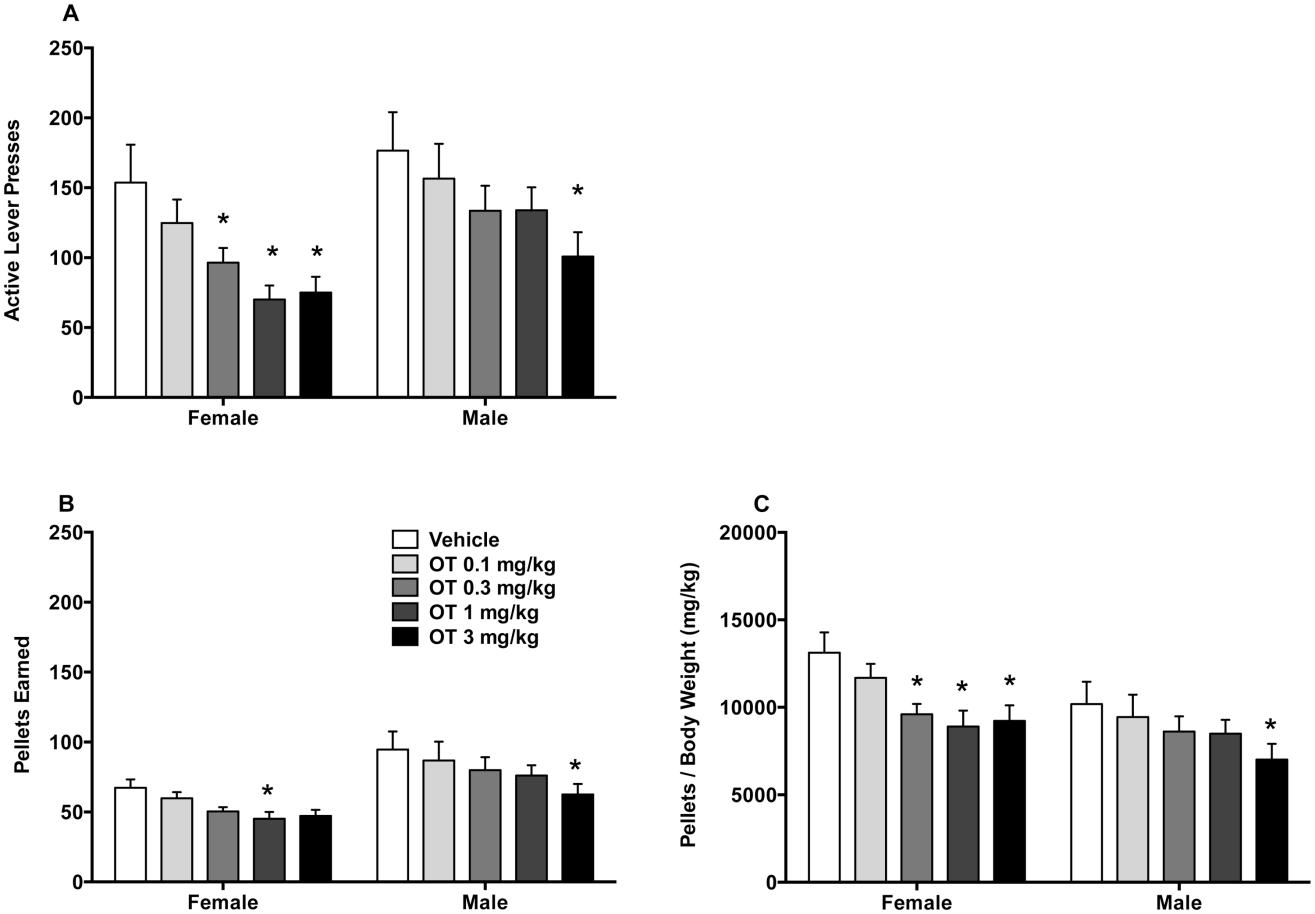
Lever presses and sucrose pellets earned during acquisition of sucrose responding following oxytocin treatment. A) Active lever presses in male and female rats. Females were more sensitive to oxytocin than males. B) Number of actual pellets earned during acquisition following oxytocin treatment. C) Adjusted pellets earned by body weights in males and females. Females were more sensitive to oxytocin than their male counterparts. *Significantly lower than same sex vehicle (p<0.05)

When responding stabilized, each rat (n=9-10) was tested on each oxytocin dose (0-3 mg/kg) in a counter balanced manner. For active lever pressing (Figure 3A), oxytocin dose dependently decreased lever responding in both sexes [dose main effect, F(4,68)=8.2, p<0.05]; however, there was not a sex difference, nor did sex interact with oxytocin dose. For females, 0.3, 1, and 3 mg/kg oxytocin decreased active lever responding, whereas the only dose that decreased responding in males was 3 mg/kg (Dunnett multiple comparisons, p<0.05). Inactive lever presses (data not shown) were reduced with oxytocin [dose main effect, F(4,68)=2.7, p<0.05]; however, follow up comparisons were not significant.

During the tests, males earned significantly more pellets than females [Figure 3B, sex main effect, F(1,17)=8.34, p<0.05] and oxytocin decreased pellets earned [dose main effect, F(4,68)=5.7, p<0.05], but there was no interaction of sex and oxytocin dose. In females, 1 mg/kg oxytocin decreased pellets earned, whereas 3 mg/kg decreased pellets in males. Oxytocin decreased sucrose earned per body weight [Figure 3C, dose main effect, F(4,68)=7.8, p<0.05], but there were no sex differences or sex × oxytocin dose interaction. Consistent with the lever responding, 0.3, 1, and 3 mg/kg oxytocin decreased pellets per body weight in females, whereas the only dose that decreased pellets per body weight in males was 3 mg/kg (Dunnett multiple comparisons, p<0.05).

### 3.3 The effect of oxytocin on sucrose conditioned cue and primed reinstatement

**Figure 4:**
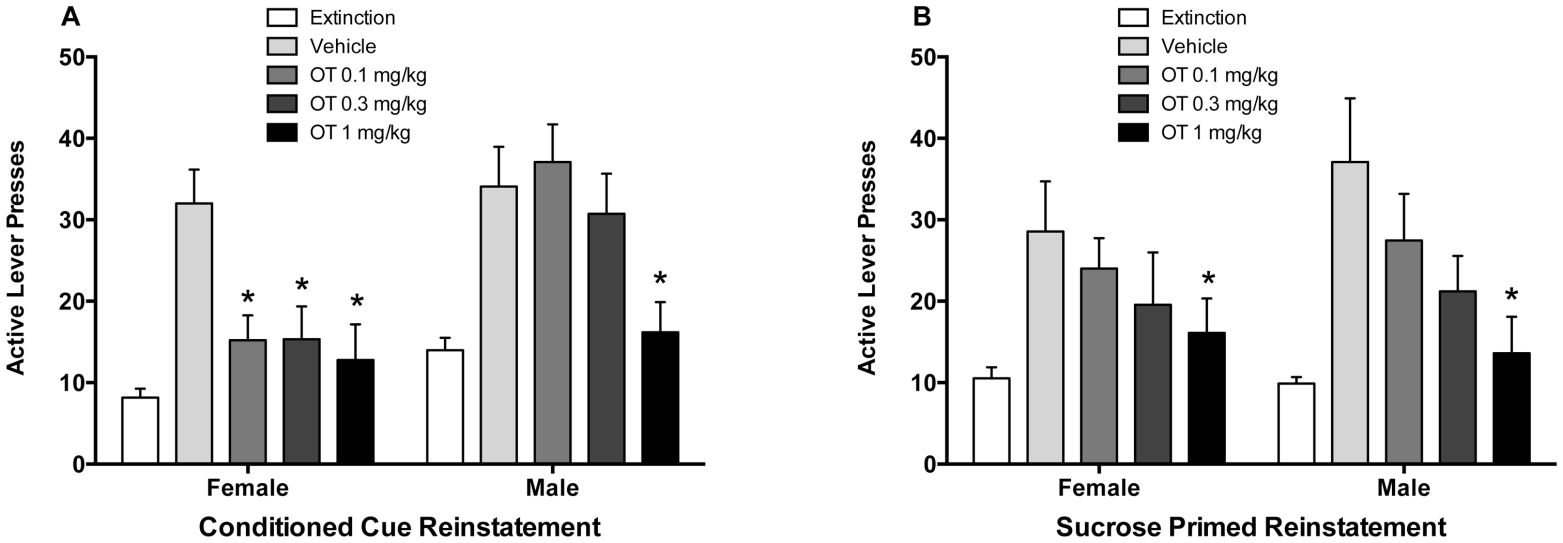
Lever presses during reinstatement tests. A) Active lever presses in conditioned cue reinstatement following oxytocin pretreatment. Females were more sensitive than males for this test. B) Active lever presses during sucrose primed reinstatement. * Significantly lower than same sex vehicle (p<0.05)

In a separate experiment, rats were tested for conditioned cue and sucrose-primed reinstatement tests following extinction. There were no differences between males and females during acquisition or extinction (data not shown), but clear sex differences emerged in response to different oxytocin doses during the presentation of sucrose-conditioned cues [Figure 4A, sex × oxytocin dose interaction, F(3,54)=2.93, p<0.05)]. Specifically, 0.1, 0.3, and 1 mg/kg oxytocin decreased active lever responding in females, whereas the only dose that decreased responding in males was 1 mg/kg (Dunnett multiple comparisons, p<0.05). In contrast, sucrose primed reinstatement was only affected by oxytocin at the highest dose in both males and females [Figure 4B, oxytocin main effect, F(3,51)=3.67, p<0.05 and Dunnett multiple comparisons, p<0.05)]. Oxytocin had no effect on inactive lever responding during either reinstatement test (data not shown).

## 4. Discussion

Here, we have established sex differences in the dose response of oxytocin on locomotor activation, lever responding for sucrose, and sucrose seeking. In fact, females were more sensitive to lower doses of oxytocin on all behavioral tests and similarities only emerged with the highest dose tested. Our findings are consistent with the notion that oxytocin regulates many of the same behaviors in males and females, but the effects are typically more profound in females (25, 26). Interestingly, the only test that females did not show an enhanced sensitivity to oxytocin pretreatment was during the sucrose primed reinstatement test. Males and females both responded in a manner consistent with our previous report that 1 mg/kg oxytocin reduced responding to a sucrose prime (12). These findings are timely given the growing interest in therapeutic treatment with oxytocin for a variety of conditions (27). Oxytocin has been suggested for treatment of autism, borderline personality disorder, anxiety and attachment disorders, weight reduction, as well as the treatment of addiction. In regards to addiction, recent findings suggest that oxytocin may have therapeutic value by blocking reward related behaviors (8).

Oxytocin impacts stimulant induced locomotor activity (28); unpublished data from our laboratory). However, it is often difficult to detect decreased activity from baseline because locomotor activity is already quite low (i.e., floor effect). Consequently, we purposely tested locomotor activity in a novel environment in order to focus on exploratory behavior early in the session. This methodology allows us to assess a time period when locomotor activity is at its highest level in non-manipulated animals. Different patterns of activity emerged between males and females, such that 0.3-3 mg/kg oxytocin decreased locomotion in females, but only the highest dose was effective in males. The most parsimonious reason for this decrease is a transient impairment of motor function. However, we previously demonstrated the 1 mg/kg oxytocin was insufficient to reduce lever responding on a progressive ratio in males or females (12), ruling out a gross loss of motor control at doses of ≤1 mg/kg. More likely, oxytocin had an increased anxiolytic effect in females because the test occurred in a novel environment, which can be used as an assessment of anxiety in rodents. In males, centrally administered oxytocin (ICV) lowered anxiety related behaviors on an elevated plus maze (29, 30). The circuitry by which oxytocin mediates anxiety is believed to be sexually dimorphic with the paraventricular nucleus and the amygdala having predominate roles in males and females, respectively (29, 31). Although beyond the scope of this study, it is possible that a sexually dimorphic role of the amygdala in oxytocin mediated anxiety and the unequal distribution of amygdala oxytocin receptors (15) contributed to our finding of increased female sensitivity to oxytocin in a novel environment.

During acquisition of sucrose taking, males showed more active lever presses and earned more pellets. However, when taking into account the smaller size of females, sucrose per kg of body weight was similar between the sexes. This distinction is important because omitting adjustments per body weight can misrepresent the behavioral output and potentially contribute to misinterpretations. During self-administration, females were more sensitive to oxytocin's ability to decrease active lever presses and the number of pellets earned (per body weight) than males. Possible causes include satiation and/or decreased salience of sucrose reward. In regards to satiation, several studies have demonstrated a link between oxytocin receptor signaling and food intake. First, mice lacking oxytocin receptors consumed larger meals than their wild type counterparts during the dark cycle (32). Second, mice with reduced hypothalamic oxytocin levels showed higher daily food intake than those with normal levels (33). Finally, centrally administered oxytocin and oxytocin agonists induced anorexia in rats, which was blocked by oxytocin receptor antagonists (34). Combined, these studies suggest that oxytocin may have influenced satiation or appetite suppression in our study. However, oxytocin failed to reduce sucrose lever pressing or break points in males and females when tested under a progressive ratio schedule, indicating that motivated sucrose seeking may not be regulated by oxytocin (12).

Female sensitivity to oxytocin may also indicate a sex specific decrease in the salience of sucrose reward. Although distribution varies according to species, oxytocin receptors are widely distributed throughout the brain (1), including regions of the mesocorticolimbic dopamine system that are critically involved in reward processing (9). In rats, receptor expression is sexually dimorphic in many areas (15), with females exhibiting lower oxytocin receptor binding densities in the posterior bed nucleus of the stria terminalus, dorsal caudate putamen, nucleus accumbens, hippocampus CA1, and medial amygdala (15). Given this lower level of centrally distributed oxytocin receptors, it is possible that lower concentrations of oxytocin saturate the available receptors in females, resulting in an increased behavioral response to the peptide. In our study, 0.3 mg/kg was sufficient to reduce locomotor activity in females. Administration of higher doses (0.3, 1, or 3 mg/kg) did not result in further decreases. Thus, higher oxytocin doses did not exert a greater effect, giving credence to the hypothesis that receptors had become saturated in females. Likewise, 0.1 mg/kg oxytocin reduced conditioned cue reinstatement to the same extent as the other test doses (i.e., 0.3 and 1 mg/kg oxytocin). Unlike cue-induced reinstatement, oxytocin reduced sucrose primed reinstatement at the 1 mg/kg dose. A possible explanation for the different results may be the distinct neuronal circuitries involved in these two forms of reinstatement, which has been demonstrated in drug self-administration studies (35, 36). For example, the amygdala is a sexually dimorphic brain region in regards to oxytocin receptor expression (15), and this brain area is critical for in drug paired-cue reinstatement, but not drug primed reinstatement (35, 36).

Only one oxytocin dose (1 mg/kg) decreased sucrose-primed reinstatement in males and females, indicating that oxytocin has a diminished impact when the primary reinforcer is present in both males and females. In contrast, oxytocin reduced cue-induced reinstatement across a range of doses in females but was only effective at one dose in males. This sex difference suggests that males and females may differ in processing conditioned associations. Such a notion is supported by sex differences in conditioned place preference for drugs (37) and in fear inhibited feeding (38). However, a more parsimonious explanation may exist. The same oxytocin doses that decreased sucrose cue reinstatement in females also decreased locomotor activity. As such, we cannot entirely rule out locomotor effects in females.

Females were tested on response criteria rather than cycle phase, producing an uneven distribution of cycle phase during the different tests and oxytocin doses. Therefore, the possibility exists that some of the reported sex differences in response to oxytocin may be due to differential regulation of oxytocin receptors, surface expression, and/or oxytocin coupling mechanisms by estrogen and progesterone. Estrogen can increase oxytocin receptor density in the ventromedial nucleus (39) and receptor affinity in the medial preoptic area of the hypothalamus (40). Moreover, the increased effect of oxytocin in females may be influenced by progesterone-induced increase of oxytocin receptor density in limbic structures (41).

Our findings reveal that females are more sensitive than males to lower doses of oxytocin in both conditioned and unconditioned behavioral responses. The oxytocin system, including receptor expression, is sexually dimorphic. These sexual dimorphisms likely contribute to the enhanced sensitivity to oxytocin expressed behaviorally in females. Together these findings suggest that the circuitry underlying these oxytocin-induced behavioral changes needs to be more fully explored. In addition, sex differences derived from this and other such studies can directly inform clinical studies that use oxytocin in treatment protocols. Specifically, current proposals by health care professionals to apply oxytocin as a treatment of autism, borderline personality disorder, anxiety and attachment disorders, and addiction should establish separate dose parameters to account for greater female sensitivity to the neuropeptide.

## Highlights

- Oxytocin impacts conditioned and unconditioned behaviors in male and female rats
- Oxytocin suppressed locomotor activity in females to a greater extent than males
- Oxytocin decreased sucrose intake and seeking greater in females relative to males
- Overall, females were more sensitive to oxytocin's behavioral effects than males
